# Hemp (*Cannabis sativa* L., Kompolti cv.) and Hop (*Humulus lupulus* L., Chinook cv.) Essential Oil and Hydrolate: HS-GC-MS Chemical Investigation and Apoptotic Activity Evaluation

**DOI:** 10.3390/ph15080976

**Published:** 2022-08-08

**Authors:** Elisa Ovidi, Valentina Laghezza Masci, Anna Rita Taddei, Jacopo Torresi, William Tomassi, Matteo Iannone, Antonio Tiezzi, Filippo Maggi, Stefania Garzoli

**Affiliations:** 1 Department for the Innovation in Biological, Agrofood and Forestal Systems, Tuscia University, 01100 Viterbo, Italy; 2 High Equipment Centre, Tuscia University, 01100 Viterbo, Italy; 3 Chemistry Interdisciplinary Project (CHIP), School of Pharmacy, University of Camerino, 62032 Camerino, Italy; 4 Circolo ARCI La Staffetta, Via Don Minzoni 29, 56011 Calci, Italy; 5 Department of Chemistry and Technologies of Drug, Sapienza University, 00185 Rome, Italy

**Keywords:** Cannabaceae, volatile compounds, secondary metabolites, cytotoxicity, apoptosis, ultrastructural changes

## Abstract

In this study, essential oils (EOs) and hydrolates (Hys) from Italian hemp (*Cannabis sativa* L. Kompolti cv.) and hop (*Humulus Lupulus* L., Chinook cv.) supply chains were chemically characterized and tested to investigate their apoptotic potential for the first time. Headspace–Gas Chromatography–Mass Spectrometry (HS-GC-MS) techniques were performed to describe their volatile chemical profile, highlighting a composition rich in terpene derivatives such as monoterpenes and sesquiterpenes among which *β*-myrcene, limonene, *β*-caryophyllene and *α*-humulene were the main constituents of EOs; in contrast, linalool, *cis*-*p*-menth-2,8-dien-1-ol, terpinen-4-ol, *α*-terpineol, caryophyllene oxide, and *τ*-cadinol were found in the Hys. The cytotoxicity activity on human leukemia cells (HL60), human neuroblastoma cells (SH-SY5Y), human metastatic adenocarcinoma breast cells (MCF7), human adenocarcinoma breast cells (MDA), and normal breast epithelial cell (MCF10A) for the EOs and Hys was studied by MTT assay and cytofluorimetric analysis and scanning and transmission electron microscopy were performed to define ultrastructural changes and the mechanism of cells death for HL 60 cells. An induction of the apoptotic mechanism was evidenced for hemp and hop EOs after treatment with the corresponding EC_50_ dose. In addition, TEM and SEM investigations revealed typical characteristics induced by the apoptotic pathway. Therefore, thanks to the integration of the applied methodologies with the used techniques, this work provides an overview on the metabolomic profile and the apoptotic potential of hemp and hop EOs and, for the first time, also of Hys. The findings of this preliminary study confirm that the EOs and Hys from *Cannabis* and *Humulus* species are sources of bioactive molecules with multiple biological effects yet to be explored.

## 1. Introduction

The genera *Cannabis* L. and *Humulus* L. belong to the Cannabaceae family in which eight more genera (*Celtis*, *Pteroceltis*, *Aphananthe*, *Chaetachme*, *Gironniera*, *Lozanella*, *Trema*, and *Parasponia* spp.) have recently been included based on phylogenetic studies [1,2].

As widely reported in the literature, the *Cannabis* genus has long been debated over the taxonomic rank of various groups and whether it is monospecific or polyspecific; it is currently believed to consist of a single species, namely *Cannabis sativa* L. [3] with subspecies each further subdivided into domestic and wild varieties [4]. *C. sativa* is a dioecious, rarely monoecious, plant with sexual dimorphism occurring at a late stage of development whereby male plants are generally taller and slimmer than female ones [5,6]. The plants are characterized by a developed taproot system and a green, straight stem, covered with hairs and branched at the top. The most recognizable organ of the plant is the palmate and incisal leaf [7]. Since ancient times, as reviewed by Bonini et al. [8], *C. sativa* has been known for its therapeutic and recreational uses. *C. sativa* is a complex plant characterized by about 500 chemical compounds, of which more than 60 are cannabinoids [9] and more than 100 are aromatic terpenes. Among cannabinoid compounds, *δ*-9-THC, CBD, CBG, *δ*-8-THC, and CBN represent the main components [10,11] and although their chemical structures are similar, their pharmacological effects can be very different. Levels of *δ*-9-THC, known for its psychotropic effects, and CBD, non-intoxicating cannabinoid, are fundamental for classifying cannabis into drug-type, marijuana varieties (high amount of THC) or non-drug-type, hemp varieties (high amount of CBD) that can be used for industrial purposes [12,13,14]. There is also another classification according to which five chemotypes are distinguished, each of which with a specific function, based on quantities and ratios of phytocannabinoids especially those of THC and CBD [15].

Generally, cannabinoids are synthesized as acidic forms then, following the drying and heating process, the acids decarboxylate generating the final neutral form [16].

The *Humulus* genus, closely related to *Cannabis* [17], comprises three species of which *H. lupulus* (hop) is widely cultured and used in brewing industry as aromatizing and flavouring agent or in medicinal research [18]. Hop is a wind-pollinated dioeciuos plant with a climbing herbaceous stem that can reach 15 m in height and is indigenous to north-temperate regions [19]. The mature female inflorescences, forming typical cones, are used in various stages of beer production and give it bitterness and aroma. Since ancient times, different uses of hop in folk medicine have been known, such as the treatment of insomnia, anxiety, spasms, cough, fever, inflammation, liver, gynecological disorders, digestion issues, and others [20,21]. The chemistry of hop is also complex; in fact, whole hop cones include several components, such as resin, essential oil, phenolic compounds, proteins, lipids, waxes, cellulose, and amino acids [22,23]. Among these, the constituents of the essential oil are those that contribute most to the aroma of beer by giving it a distinctive flavor. On the other hand, hop petals are characterized by the presence of proteins, carbohydrates, and polyphenols [24].

In this work, with the aim to describe the chemical composition and evaluate the apoptotic activity of Kompolti hemp and Chinook hop cultivars, their essential oils (EOs) and hydrolates (Hys) were investigated. Up to now, no contribution has been reported on hop and hemp hydrolates regardless of the type of variety studied, both in terms of chemical composition and their biological activity.

## 2. Results

### 2.1. EOs Density and Refractive Index Determination

Hop and hemp EOs density was measured at 20 °C through an oscillating U-tube density meter (DA-100M, Mettler Toledo, Greifensee, Switzerland). The refractive index of EOs was calculated at 20 °C using an Abbe refractometer (NAR-1T LIQUID, Atago Co., Ltd., Tokyo, Japan). The hop EO density and refractive index were 0.88 g/cm^3^ and 1.485 g/cm^3^, respectively, while a density of 0.88 g/cm^3^ and refractive index of 1.489 g/cm^3^ were found for hemp EO.

### 2.2. Chemical Analyses of Chinook Hop and Kompolti Hemp Hys and EOs

The chemical composition of the EOs was obtained by GC/MS technique. In total 53 compounds were identified of which 28 were in hop EO and 31 in hemp EO (Table 1). In hop EO, the sesquiterpenes (79.7%) prevailed over the monoterpenes contrary to the hemp EO, where the monoterpene fraction was more abundant. Qualitative and quantitative differences were found between the two EOs. In detail, humulene (38.0%) followed by *β*-caryophyllene (12.2%), *δ*-cadinene (9.4%), and *τ*-cadinol (4.5%) were the major sesquiterpenes detected in hop EO. In contrast, in hemp EO, *β*-caryophyllene (22.2%) prevailed over humulene (6.5%). Further, among monoterpenes, limonene (27.9%), *β*-myrcene (12.4%), *α*-pinene (8.6%), and terpinolene (6.2%) were the principal component in hemp EO while *β*-myrcene (9.4%) was the main one in hop EO.

**Figure 1:**
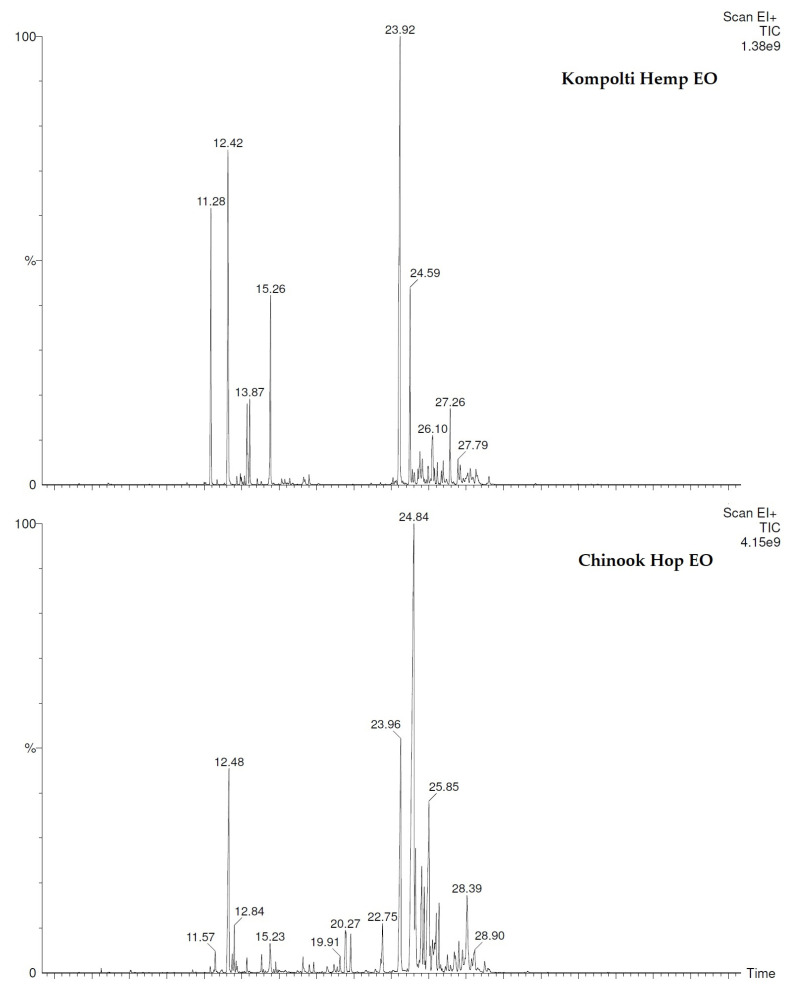
Chromatograms of hemp and hop EOs.

A series of minor compounds have only been detected in one oil or another. Among them, some terpenes with a percentage value greater than 1% such as linalool (1.3%), (*Z*)-methyl-geranate (1.2%), *α*-copaene (1.8%), *γ*-muurolene (3.6%), *α*-selinene (2.1%), and *α*-eudesmol (1.4%) were characteristic of the hop EO chemical profile while *cis*-*β*-ocimene (2.1%) and *β*-maaliene (2.3%) were found in hemp EO. The chromatograms of both EOs were reported in Figure 1.

**Figure 2:**
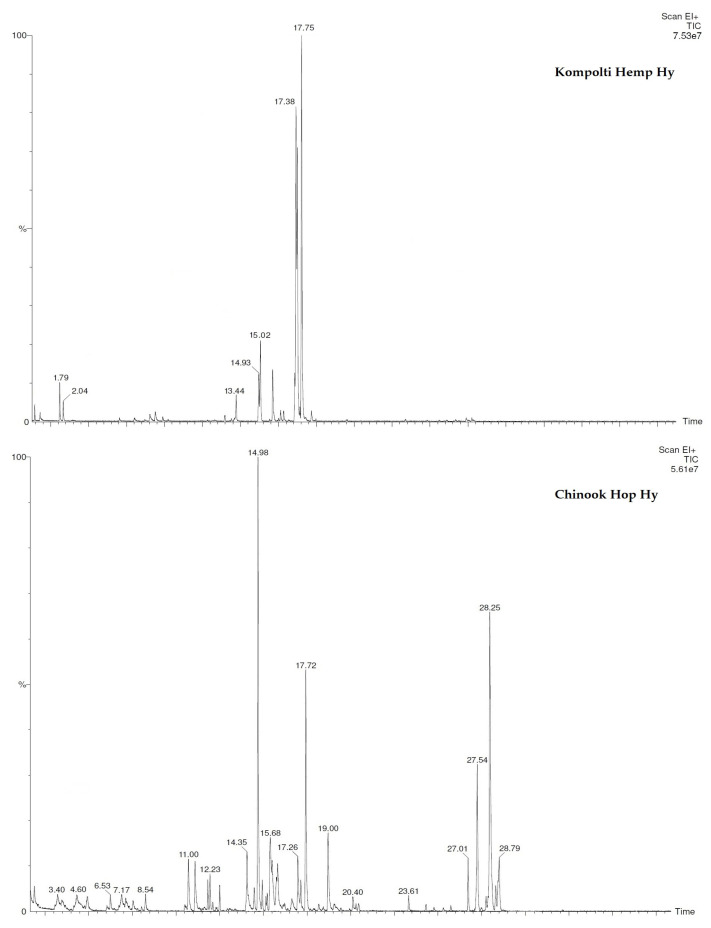
Chromatograms of hemp and hop Hys.

The volatile composition of hop and hemp Hy carried out by HS-GC-MS technique highlighted the presence of 29 compounds, 20 in the hop Hy and 14 in that of hemp (Table 2). The chemical profile of the two Hys differed in that *τ*-cadinol (22.9%) followed by linalool (21.2%) and *α*-terpineol (12.7%) were the most abundant components of hop Hy, while *α*-terpineol (31.2%), *cis*-*p*-mentha-2,8-dien-1-ol (25.7%), and terpinen-4-ol (18.1%) were the ones in hemp Hy. Only five components, namely hexanal, linalool, isoborneol, terpinen-4-ol, and *α*-terpineol were detected in both Hys; moreover, sesquiterpenes such as caryophyllene oxide, *τ*-cadinol, germacrene D, and *α*-eudesmol, with percentage values ranging from 1.2 to 22.9%, were characteristic only of the hop Hy. In general, the monoterpene content exceeded the sesquiterpene one. The chromatograms of both Hys are reported in Figure 2.

### 2.3. Cytotoxicity Determination of EOs and Hys Using MTT Assay

The cytotoxic activity of hemp and hop EOs and Hys against different human cells lines was examined by dose-dependent approach at 48 h of treatments. In Table 3, we reported the concentrations of EOs and Hys able to reduce the 50% of cell viability (EC_50_). The HL60 cells were shown to be the most sensitive to the treatments, with EC_50_ values of 10.49 × 10^−3^ mg/mL and 38.92 × 10^−3^ mg/mL after 48 h of hemp and hop EOs incubation, respectivetly. The hemp EO was more active toward the neuroblastoma cells (SH-SY5Y), with an EC_50_ value of 95.87 × 10^−3^ mg/mL, than that of hop which had a value of 315.60 × 10^−3^ mg/mL. Both EOs were also quite active against the two breast cancer cells, MCF7 and MDA (241.10 × 10^−3^ mg/mL and 230.17 × 10^−3^ mg/mL for hemp EO, 237.20 × 10^−3^ mg/mL and 317.51 × 10^−3^ mg/mL or hop EO, respectively).

**Figure 3:**
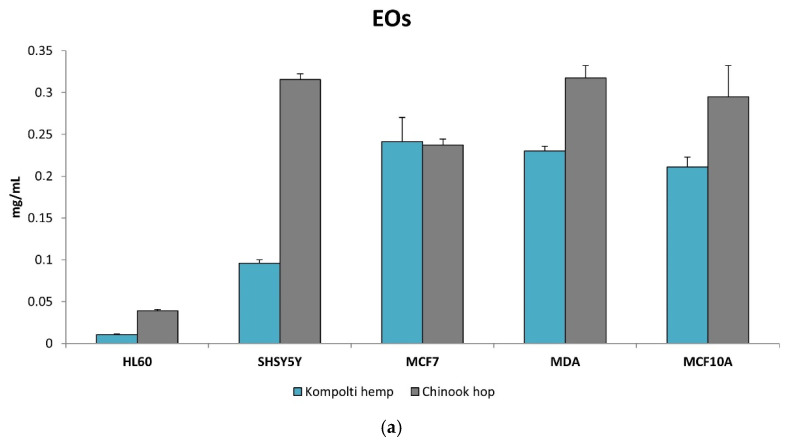

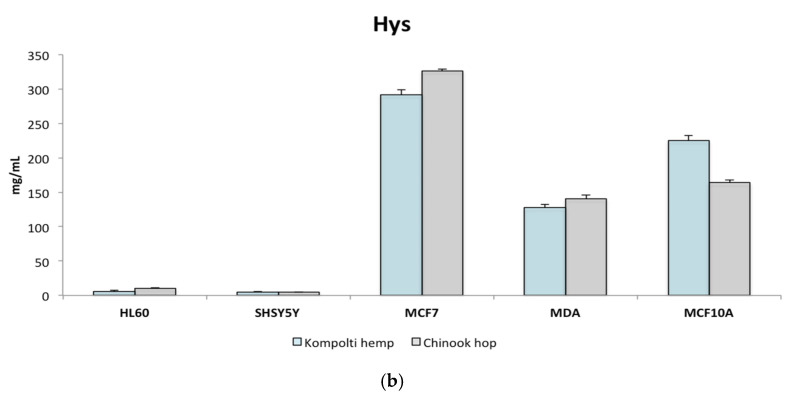
The EC_50_ values of EOs (**a**) and Hys (**b**) from Kompolti hemp and Chinook hop. The values are expressed as mg/mL ± standard error mean (sem).

Concerning hemp and hop Hys, EC_50_ values were 6.05 mg/mL and 4.90 mg/mL, respectively. Likewise, low EC_50_ values were obatined for the treatment of SH-SY5Y cells (4.90 mg/mL for hemp and 10.46 mg/mL for hop). Higher EC_50_ values for the corresponding treatment on MCF-7 and MDA cells were obtained (Table 4). Figure 2 shows the bar graphs of the EC_50_ values of EOs (Figure 3a) and Hys (Figure 3b) obtained from Kompolti hemp and Chinook hop.

As reported in Table 3 and Table 4, the EC_50_ values obtained from the treatment against the normal human MCF10A cell line were used to define the Selectivity Index (SI). For EOs (Table 3), the lowest SI value was observed for human leukemic cells (20.11 × 10^−3^ mg/mL for *C. sativa* EO and 7.57 × 10^−3^ mg/mL for *H. lupulus* EO). In the case of Hys, the lowest SI values were observed for *C. sativa* (37.25 mg/mL and 45.99 mg/mL) and for *H. lupulus* (15.67 mg/mL and 36.53 mg/mL), against leukemia and neuroblastoma cells, respectively (Table 4).

### 2.4. Flow Cytometry Analysis

**Figure 4:**
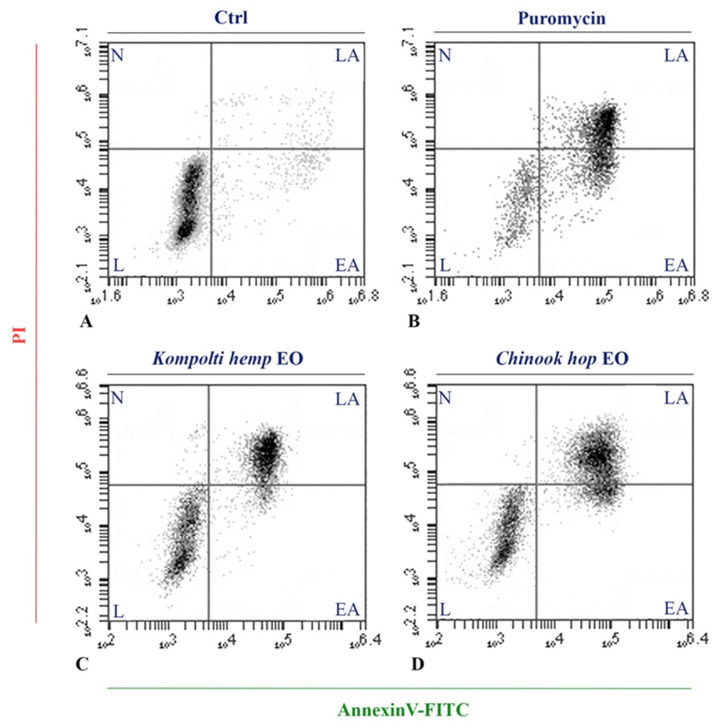
Flow cytometric analysis of the Kompolti hemp and Chinook hop EOs treatments on HL60 cells for apoptosis determination using AnnexinV-FITC/PI staining. (**A**): Ctrl; (**B**): Puromycin; (**C**): *Kompolti* hemp EO; (**D**): *Chinook* hop EO. L: live cells; EA: early apoptotic cells; LA: late apoptotic cells; N: necrotic cells.

To assess the cell death mechanism occurred by the EOs treatments, the reduced cell viability of HL60 cells was further investigated by Annexin/FITC and propidium iodide in flow cytometry analysis. Each treatment was carried out using the corresponding EC_50_ value for 24 h and live (double negative), early apoptotic (EA, AnnexinV-positive/PI-negative staining), late apoptotic (LA, double positive), and necrotic cells (PI positive) per-centages were defined. The apoptotic rates, resulting from the sum of the percentages of AnnexinV-positive/PI-negative and double-positive stained cells, are shown in Figure 4.

**Figure 5:**
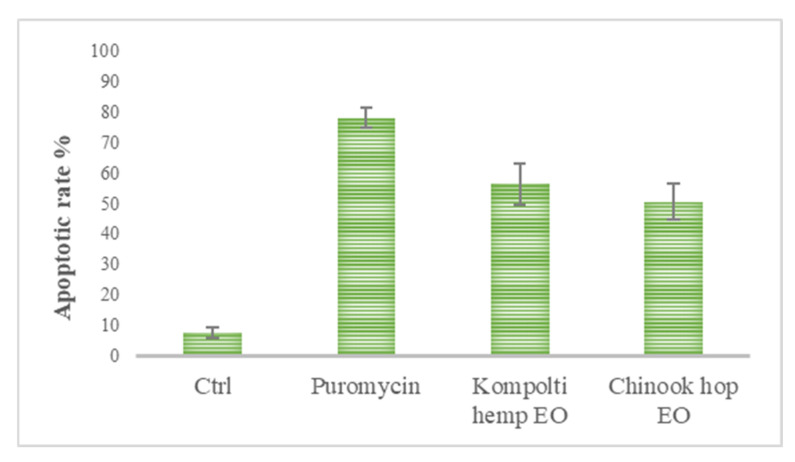
Bar graph of apoptotic rates obtained from the sum of the percentages of EA and LA cells in flow cytometric analysis using AnnexinV-FITC/PI staining. The values are expressed as % ± standard error mean (sem).

In ctrl (control) cells, the apoptotic rate was 7.87% ± 1.68% whereas in puromycin treated cells apoptotic cells was 78.05% ± 3.47%. After 24 h of treatment with hemp and hop EOs, a high level of Annexin V-positive/PI-negative staining and double-positive staining were recorded (56.32% ± 6.78% and 50.47% ± 5.90%, respectively) (Figure 5).

Treatments with Hys of both plants did not determine an increase of the number of EA and LA cells (data not shown).

### 2.5. Ultrastructural Investigations by Transmission and Scanning Electron Microscopy

To define the effects of treatments with Kompolti hemp and Chinook hop EOs, SEM and TEM investigations were conducted on HL60 cells treated for 4 h, as well as on untreated and puromycin-treated cells, the latter as a hallmark of apoptosis.

**Figure 6:**
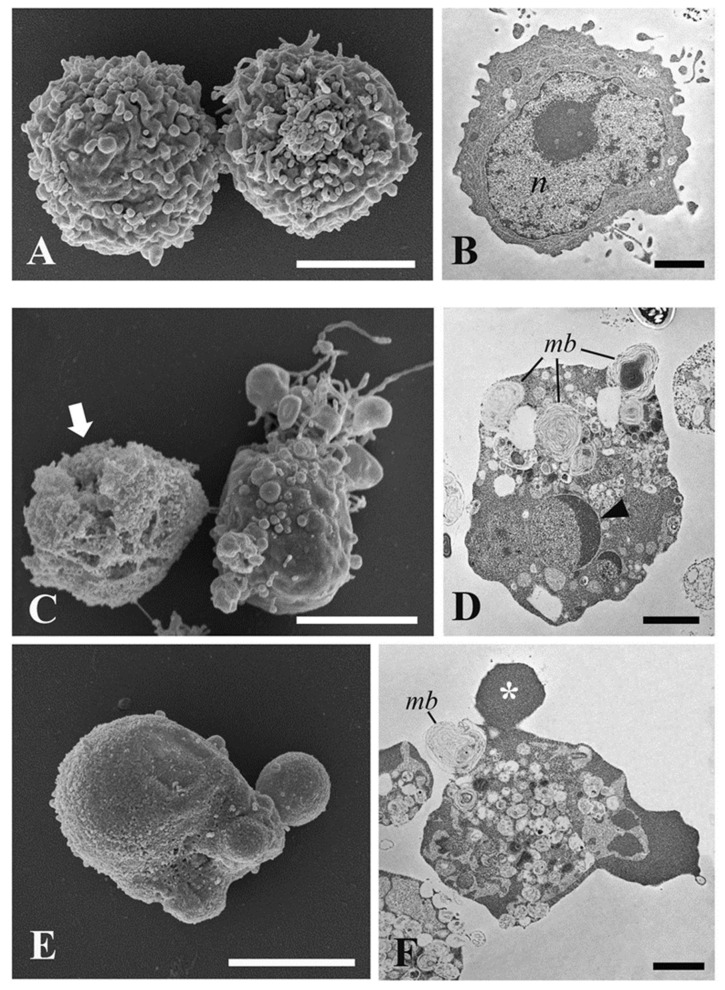
SEM and TEM investigations on HL60 cells. (**A**) SEM micrograph of untreated HL60 cells; (**B**) TEM micrograph of untreated HL60 cells; (**C**,**E**) SEM micrograph of puromycin-treated HL60 cells for 4 h; (**D**,**F**) TEM micrograph of puromycin-treated HL60 cells for 4 h. *n*, nucleus; *mb*, multilamellar bodies; arrow, dead cell; asterisk, fragmented nucleus. Bars = (**A**,**C**,**E**) 5 μm; (**B**,**D**,**F**) 2 μm.

In Figure 6A, the untreated cells show their typical morphology with a round shape and a cell membrane characterized by the presence of numerous regularly distributed cell protrusions. TEM analysis of untreated HL60 cells revealed its typical promyelocytic ultrastructure in which the nucleus occupies most of the cell volume and the cytoplasm is organized with the characteristic cellular compartments (Figure 6B). The puromycin-treated cells showed the morphological features induced by the apoptotic mechanism, such as shrinkage of the cell, a smoother surface with round-shaped structures due to an intense surface blebbing (Figure 6C,E). Dead cells were also observed (Figure 6C).

**Figure 7:**
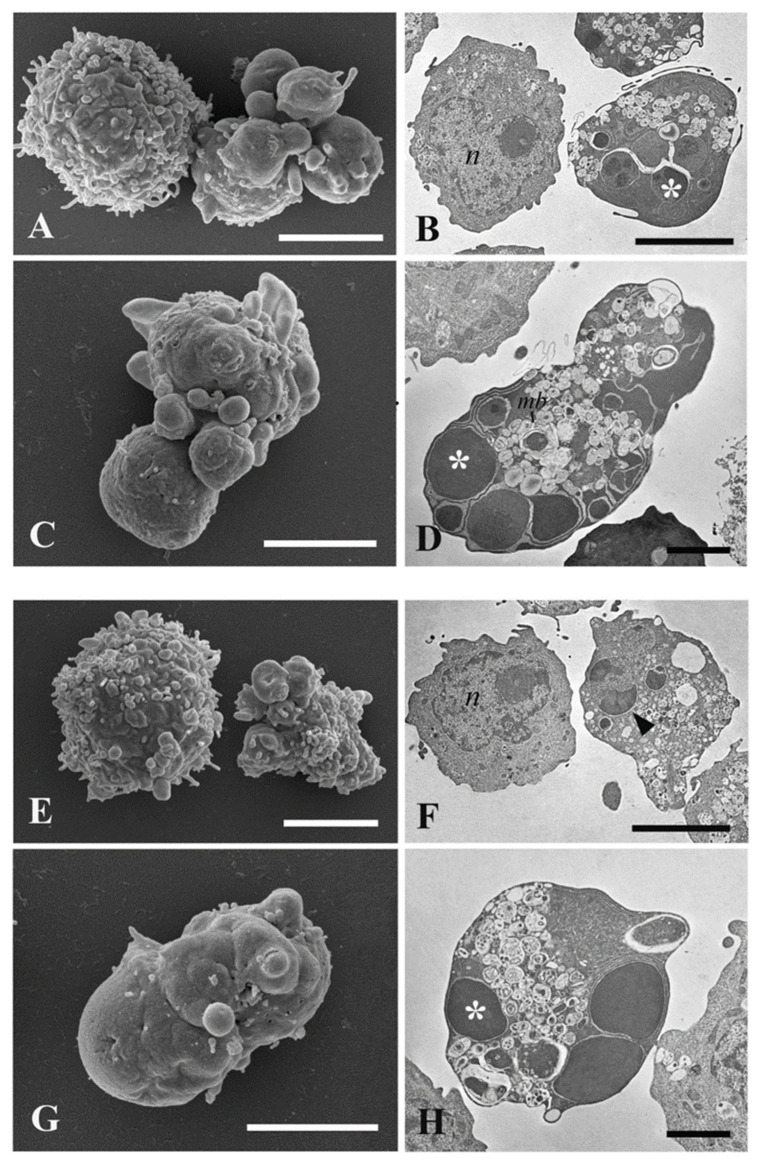
SEM and TEM investigations of ultrastructural changes induced by Kompolti hemp and Chinook hop EO treatments on HL60 cells. (**A**–**D**) Micrograph of hemp EO treated HL60 cells. (**E**–**H**) Micrograph of hop EO treated HL60 cells. *n*, nucleus; arrow, dead cell; *mb*, multilamellar bodies; asterisk, fragmented nucleus. Bars = (**A**–**C**,**E**–**G**) 5 μm; (**D**,**H**) 2 μm.

By TEM analysis, puromycin treatment resulted in changes in cell ultrastructure, such as cytoplasmic and nuclear condensation, nuclear fragmentation (karyorrhexis), presence of multilamellar bodies, and cellular blebs. The pyknotic nucleus appeared like a half-moon, a typical feature of an apoptotic cell (Figure 6D,F). Apoptotic characters were clearly found in the cells treated with hemp (Figure 7A–D) and hop EOs (Figure 7E–H). In hemp EO treatment, in addition to live cells with a round shape and a regular surface, cells with apoptotic blebs and swelling were found (Figure 7A,C).

TEM analysis revealed a typical apoptotic ultrastructure and the presence of fragmentation of the cytoplasm and nucleus, inclusions and multilamellar bodies surrounded by cell membranes; cell blebs were clearly evident (Figure 7B,D). Likewise, hop EO determined a cell volume shrinkage and a cellular blebbing (Figure 7E–H). Pycnotic and fragmented nucleus and condensed cytoplasm were observed.

## 3. Discussion

The chemical composition of hemp and hop EOs and Hys was obtained by application of HS-GC-MS techniques. The obtained results highlighted a composition rich in sesquiterpenes for both EOs.

EO from *C. sativa* is a complex mixture of volatile compounds made up of more than 100 terpene derivatives [25]. Regarding Kompolti variety EO composition, in our study, limonene and β-caryophyllene were the most abundant components as also confirmed by other authors [26,27]. Compared to those conducted on the Kompolti variety, a greater number of reports concerning studies on other hop varieties such as Futura 75, Carmagnola, and Uso 31, whose compositions are characterized by an abundance of sesquiterpenes [28,29], are present. Nissen et al. [30] also investigated Carmagnola and Futura varieties but in contrast, they found a composition rich in monoterpenes with β-myrcene as the most abundant constituent in both EOs. Some studies attributed the variability of the hemp EOs chemical composition and its quality to more factors, such as the distillation technique and extraction time, the processing of plant material, the environmental factors, and cultivar type [27,31,32,33,34].

Moreover, hop EO has a complex composition with a large number of volatile compounds belonging to different chemical classes that produce the distinct waves of scent for that type of hops according to their concentrations and interactions [35,36]. In particular, a recent review provides a number of insights into the various factors that can influence the presence of the volatiles in the hop EO which contribute to defining the aroma and flavor profile of beer [37]. Previous studies conducted on EOs obtained from wild hops grown in eastern Lithuania [38,39] revealed a complex composition by identifying up to 120 compounds among which α-humulene (11.1–33.4%), *β*-myrcene (15.7–21.1%), and *γ*-elemene (14%) were the most representative components in the different hop varieties investigated. In general, the monoterpene compounds such as *β*-myrcene and sesquiterpenes including *α*-humulene and *β*-caryophyllene are the most relevant compounds of hop EOs obtained from cones [40,41]. *α*-Humulene (4.87–15.26%) and *τ*-cadinol (6.83–28.06%) were found to be the major components in five varieties investigated by Kowalaska and colleagues [42]. However, the diversity of composition between the different hop EOs, as with other EOs, depends on the varietal characteristics [43,44] and several intrinsic and extrinsic factors. Among these, the geographical origin, the different cultivars, the climatic and soil conditions, and the variability of the secondary metabolism can be decisive in defining the profiling [39,45,46]. The differences are also due to the presence of parasites [47,48] and the use of pesticides, fungicides [49], and fertilizers whose presence also may affect the EO yield [47]. Equally important are the extraction methods used to isolate the EO [50,51], the method of storage and packaging after harvesting, and the temperature applied for drying which must not be too high to avoid oxidative degradation [52].

In our study, the evaluation of the EOs and Hys cytotoxic activity was performed against several cancer cell lines, i.e., human acute promyelocytic leukemia cells, human neuroblastoma cells, human metastatic mammary adenocarcinoma cells, human mammary adenocarcinoma cells, and a normal mammary epithelial cell, by means of the cytotoxic MTT assay. For the EOs, cytofluorimetric analysis and TEM and SEM investigations were also performed.

The most sensitive cell line to the EOs treatments was the HL60 leukemia cell line which showed an EC_50_ value of 10.49 and 38.92 × 10^−3^ mg/mL for hemp and hop EO, respectively. An EC_50_ value of 95.87 × 10^−3^ mg/mL was obtained for SH-SY5Y neuroblastoma cell as the result of the treatment with hemp EO. A moderate activity of the two EOs was also obtained for the other studied cancer cell lines (EC_50_ from a minimum of 237.20 to a maximum of 317.51 × 10^−3^ mg/mL). The hemp EO was highly selective for the HL60 cell line compared to the normal MCF-10A cells with the SI value > 10 (SI = 20.11). Although the hop EO showed a low EC_50_ value for HL60 cell line, it was not considered selective being cytotoxic for the normal cell line (SI = 7.57).

The Hys of hemp and hop were less active than the corresponding EOs. However, their cytotoxic activity was interesting in comparison to the Hys biological activity of other plant species [53,54] as the EC_50_ values were rather low (EC_50_ values from 4.49 to 326.76 mg/mL). Furthermore, the SI values for HL60 and SH-SY5Y were above 10 and thus both Hys could be considered selective. In contrast, for the adenocarcinoma cell lines, the SI values of both EOs were below 10 and they were not considered selective.

The cytotoxic activity of extracts and EOs from different varieties of *C. sativa* leaves, inflorescences, and seeds were observed on different cancer and normal cell lines [55,56,57,58,59,60]. Only one recent paper reported the cytotoxic activity of the Kompolti hemp EO variety on more human cancerous cell lines showing significant effects although with a lower tolerability in noncancerous cells [61].

To determine the type of cell death after EO treatment, HL60 cells, for which EC_50_ values were very low, were further studied. By means of cytofluorimetric analysis, an induction of the apoptotic mechanism was evidenced with an apoptotic rate of 56.32% for hemp EO and 50.47% for hop EO after treatment with the corresponding EC_50_ dose. In addition, TEM and SEM investigations were performed and comparison with puromycin-treated cells revealed typical characteristics induced by the apoptotic pathway. Both the EOs determined an apoptotic morphological change such as extensive condensation of the chromatin, cytoplasmic shrinkage, blebbing of the plasma membrane, and the formation of apoptotic bodies [62].

In our investigation, limonene and *β*-caryophyllene were found to be the most abundant constituents of the Kompolti hemp EO and they have been shown to exert a chemopreventive activity and limit tumor growth and angiogenesis and induce apoptosis in several cancer cell models [63,64,65]. Furthermore, *α*-humulene, the most abundant component in Chinook hop EO, has also been reported to have anticancer and apoptotic inducing activities [66].

On the other hand, to the best of our knowledge, the apoptotic induction activity of *H. lupulus* EO had not been investigated until now. Finally, in the scientific literature, the description of the phytochemical composition of the hop and/or hemp Hys is missing. Therefore, in this study, in addition to evaluating the volatile profile of EOs, an investigation on the metabolomic profile of the respective hydrolates was carried out. The latter, performed by HS-GC-MS technique, represents the novelty of this work as well as the evaluation of the cytotoxic activity on both extractive products, also obtaining very promising results.

## 4. Materials and Methods

### 4.1. Materials

Thiazolyl Blue Tetrazolium Bromide (MTT) was from Sigma-Aldrich (Darmstadt, Germany).

### 4.2. Plant Material

Hops subjected to this study were the Chinook variety, grown in San Nicandro Garganico (Foggia, Italy), by the Agricola Vocino Farm in 2021.

The choice of this variety of hop is linked to its high content of α-acids (greater than 10%), which allow the plant to resist extreme abiotic events such as heat and drought typical of the growing area, but also biotic such as attacks from the downy mildew “Fusarium canker”.

The plants were positioned with about 1 m between one another, and between the individual rows, the distance maintained was about 3 m. Therefore, the density per hectare was about 3000 plants for a total of 150 plants in three rows. Irrigation was carried out by drop without resorting to any treatment or chemical fertilization while the tillage was mostly manual. The manual harvesting of the plants was carried out towards the end of August; over a period of 15–20 days, at least 2/3 collections were carried out. The drying of the hop cones was performed in a natural way, inside a dark room in order to protect hop from the sun’s rays, ventilated (in a natural or artificial way), and possibly with humidity control (about 50% RH). After about 5 days, the hops were vacuum-packed and stored in a cool and dry place. In these conditions, the hop inflorescences are usable for about 24 months.

The Kompolti hemp variety was grown in Torre del Lago, Lucca, Italy, by the Carmazzi Farm in 2019. This hemp variety is of Hungarian origin and is characterized by a low/no THC content and medium/low CBD content (THC: <0.2% CBD: 2.0–10.0%).

The sowing was done in April in a thermo-controlled greenhouse and under LED lamps. The plants were arranged at about 1.5 m from each other, maintaining a distance of 4 m between the individual rows. Subsequently, the seedlings were transplanted into 8 L pots and kept in the greenhouse. At the beginning of June, when the plant developed a suitable root system, it was transplanted in the open field. The soil was set aside during the winter and prepared with a superficial mechanical processing so as not to compromise the microbiome, which is important for the rooting of the plant itself.

The apical topping technique was applied to limit vegetative growth and to increase the production of apical and lateral inflorescences. Drip irrigation was performed throughout the vegetative phase and mulched with biodegradable corn cloth without resorting to any phytochemical treatment. The collection of the inflorescences, carried out early in the morning to preserve the aromatic component of the inflorescences, began at the end of August until the beginning of September and no presence of parasites was ever observed. After harvesting, the inflorescences were manually cleaned of the leaves and then placed in vacuum packs and stored in the freezer at a temperature of −20 °C.

### 4.3. Hydrodistillation (HD)

Chinook hop EO and Hy were obtained by hydrodistillation (HD). In particular, 1.4 kg of dried plant material were distilled in two round flasks of 10 L capacity, each containing 700 g of hop and 6 L of distilled water. Hop inflorescences were soaked in distilled water overnight, and then HD was performed for 4 h using a Clevenger-type apparatus and a heating mantle system Falc MA (Falc Instruments, Treviglio, Italy). Hop EO and hydrolates were collected separately and kept at 4 °C until further analysis. The EO yield accounted for 0.37% w/w.

### 4.4. Moisture Content Evaluation of Frozen Hemp

In order to determine the water content, 3 homogeneous samples of Kompolti frozen inflorescences were heated at 100 °C on a thermo balance (Scaltec SMO 01, Burladingen, Germany), and an average value of 60.6% was obtained.

### 4.5. Microwave-Assisted Extraction (MAE)

Kompolti hemp EO and Hy were obtained through the advanced microwave extraction system Milestone ETHOS X (Milestone, Italy). A total of 3.3 kg of frozen plant material was left to defrost for 30 min, and then subjected to 3 MAE runs. In each of them, 1.1 kg of inflorescences was processed with 1.3 L of distilled water, in a glass reactor of 12 L capacity, and placed within the microwave reactor, operating at 2.45 GHz. The microwave power was set at the maximum value delivered by the instrument, namely 1800 W, and the extraction time was 3 h. A stainless-steel Clevenger-type apparatus above the system, and a Chiller Smart H150-2100S by Labtech srl (Sorisole, Bergamo, Italy), which kept water temperature at 8 °C, enabled the distillation process. Kompolti EO and hydrolate were separated and stored in glass vials at 4 °C before analysis. The EO yield was estimated on dry matter (*w*/*w*) and was 0.35%.

### 4.6. Headspace–Gas Chromatography–Mass Spectrometry (HS-GC-MS)

The volatile profile of the EOs and HYs vapor phase was investigated using a Perkin-Elmer Headspace Turbomatrix 40 (Waltham, MA, USA) autosampler connected to GC-MS. The operative conditions were performed as previously described [67]. About 1 mL of each EO and 2 mL of each Hy were placed separately in 20 mL vials sealed with headspace PTFE-coated silicone rubber septa and caps. The identification and quantifications of compounds were performed as described in Section 4.7.

### 4.7. Gas Chromatography–Mass Spectrometry (GC–MS) Analysis

To characterize the chemical composition of all samples, a Clarus 500 model Perkin Elmer (Waltham, MA, USA) gas chromatograph coupled with a mass spectrometer and equipped with a FID (flame detector ionization) was used. Chromatographic separation was performed using a Varian Factor Four VF-1 capillary column and gas carrier was He at flow rate of 1.0 mL/min in constant flow mode. The GC operative conditions followed Ovidi et al. [68]. The volatile separated compounds were identified by matching their mass spectra with those stored in the Wiley 2.2 and Nist 02 mass spectra libraries database and by comparison of their linear retention indices (LRIs), relative to C_8_–C_30_ n-alkanes analyzed under the same conditions, with those available in the literature. Relative concentrations of individual compounds were expressed as percentage of the relative peak area to that of the total peak area without the use of an internal standard and any factor correction. All analyses were carried out in triplicate.

### 4.8. Cell culture and Maintenance

To evaluate the effect of EOs and HYs on cell viability, five different cell lines were used: human acute promyelocytic leukemia cells (HL60, ATCC^®^ CCL-240), human neuroblastoma cells (SH-SY5Y, ATCC^®^ CRL-2266), human metastatic adenocarcinoma breast cells (MCF7, ATCC^®^ HTB-22™), human adenocarcinoma breast cells (MDA, ATCC^®^ MB-231), and normal breast epithelial cell (MCF10A. ATCC^®^ CRL-10317™).

The HL60 were cultured in RPMI-1640 supplemented with 10% FBS, 1% glutamine, and 1% penicillin-streptomycin. The SH-SY5Y, MCF7, and MDA cells were cultured in DMEM F-12 supplemented with 10% FBS, 1% glutamine, and 1% penicillin-streptomycin. The MCF10A were maintained in DMEM F-12 medium supplemented with 100 ng/mL cholera toxin, 20 ng/mL epidermal growth factor (EGF), 0.01 mg/mL insulin, 500 ng/mL hydrocortisone, 5% Horse Serum (HS), 1% glutamine, and 1% penicillin-streptomycin; before being seeded for the viability assay, the culture medium was deprived of EGF and the HS was reduced to 2%. All the cell lines were maintained at 37 °C in a humidified 5% CO_2_ condition. All experiments were performed with the cells in their logarithmic growth phase.

#### 4.8.1. Cytotoxicity Assay

The cytotoxicity assay was performed using the MTT assay as described by Garzoli et al. [69]. The cells were seeded in 96 flat-bottomed tissue culture plate at seeding concentrations of 2 × 10^5^ cells/mL for HL60 and SH-SY5Y cells, and 1 × 10^5^ cells/mL for the MCF7, MDA, and MCF10A cells. The plates were incubated in a humidified incubator with 5% CO_2_ at 37 °C for 24 h to allow the cells to attach to the bottom of the wells. A stock solution of EOs and DMSO at 50% was prepared to improve the solubility and bioavailability of essential oils. Subsequently, 10 two-fold different concentrations were added to the media, from 4 to 0.008 mg/mL for the EOs and from 500 to 1 mg/mL for the Hys. DMSO and H_2_O were used as solvent controls. Vinblastine was used as positive control. A dose-dependent treatment for 48 h was carried out to evaluate the cytotoxicity. Following each treatment, the media containing the compounds were aspirated and 100 µL of 0.5 mg/mL of 3-(4,5dimethylthiazol-2-yl)-2,5-diphenyltetrazolium bromide (MTT) was added to each well. After 3 h, the MTT solution was removed and 100 µL of DMSO was added to solubilize the purple-colored formazan product. The absorbance was read at 595 nm by a Tecan Sunrise™ UV–Vis spectrophotometer. The absorbance reading was corrected to background absorbance, and average reading was taken from three independent experiments. The cell viability expressed as percentage was calculated as follows:

$$\;\mathrm{Cell}\;\mathrm{viability}\;\%\;=\frac{\mathrm{Average}\;\mathrm{absorbance}\;\mathrm{of}\;\mathrm{treated}\;\mathrm{cells}}{\mathrm{Average}\;\mathrm{absorbance}\;\mathrm{of}\;\mathrm{untreated}\;\mathrm{cells}\;\left(\mathrm{control}\right)}\times 100$$

The results were analyzed using GraphPad Prism version 8.0.2 for Windows (GraphPad Software, La Jolla, CA, USA). The half-maximal concentration (EC_50_) for each biological replicate was determined using log-response curve. Overall, EC_50_ was calculated from the average EC_50_ of three independent biological replicates.

#### 4.8.2. Selective Index

The Selective Index (SI) is defined as the selectivity of a tested substance against cancerous cells and a value higher than 10 belongs to a selected potential sample that can be further investigated [70].

It was calculated as follows:

$$\mathrm{Selective}\;\mathrm{index}\;\mathrm{of}\;a\;\mathrm{tumor}\;\mathrm{cell}\;\mathrm{line}\;=\frac{\mathrm{EC}50\;\mathrm{of}\;\mathrm{MCF}10A\;\mathrm{cell}\;\mathrm{line}}{\mathrm{EC}50\;\mathrm{of}\;a\;\mathrm{tumor}\;\mathrm{cells}\;\mathrm{line}}\times 100$$

### 4.9. Flow Cytometry

To determine the apoptotic induction activity of the EOs of Kompolti hemp and Chinook hop, a flow cytometry analysis was performed on HL60 cells using the Annexin V-FITC and PI apoptosis kit (eBioscience., San Diego, CA, USA). HL60 cells were plated at a density of 5 × 10^5^ cells/mL on a six-well plate. After 24 h incubation, the cells were treated with EOs at the EC_50_ concentration.

After 24 h, the cells were stained with an Annexin V-FITC conjugate and PI, and the emitted fluorescence was analyzed using a flow cytometer (BD Accuri C6 Plus, BD Italy S.p.A, Milano, Italy). A total of 1 × 10^4^ events per sample were acquired and the obtained “dot plot”, in which each point corresponds to a single event, was recorded. The experiments were repeated three times.

### 4.10. Scanning and Transmission Electron Microscopy

For scanning and transmission electron microscopy investigations, HL60 cells were seeded at a density of 1 × 10^6^ cells/mL in a 10 mm petri dish and incubated for 24 h under appropriate conditions before adding treatments with hemp and hop EOs (EC_50_ values). After 24 h of treatment, the cells were fixed with 4% paraformaldehyde and 5% glutaraldehyde (pH 7.2) in 0.1 M cacodylate buffer for 1 h at 4 °C (Karnowsky) and then post-fixed in 1% osmium tetroxide in cacodylate buffer for 1 h at 4 °C. A stepwise ethanol series was used to dehydrate the samples.

For scanning electron microscopy (SEM), cells were dried by the critical point method using CO_2_ in a Balzers Union CPD 020 and were coated with gold in a Balzers MED 010 unit. Observations and micrographs were performed with a JEOL JSM 5200 electron mi-croscope (Jeol Ltd., Tokyo, Japan).

For transmission electron microscopy (TEM), the dehydrate samples were embedded in an Epon mix resin. Thin sections (50–70 nm) were obtained with Reichert Ultracut (Leica Mi-crosystems, Wetzlar, Germany) and LKB Nova ultramicrotome (LKB Vertriebs GmbH, Vienna, Austria) using a diamond knife, collected on copper grids, stained with uranyl acetate and lead citrate, and observed with a JEOL 1200EX II electron microscope (Jeol Ltd., Tokyo, Japan).

## 5. Conclusions

This paper aims to enrich the current state of knowledge on the potential of EOs and Hys from two singular industrial crops such as hop and hemp which have so far been less investigated than others.

Chemical analyses highlighted a rich volatile composition and the obtained results from performed biological assays showed the cytotoxic properties, on multiple human cell lines, of both Kompolti and Chinook EOs and, unexpectedly, also of the respective Hys. In particular, an induction of the apoptotic mechanism was evidenced for HL60 cells; in fact, TEM and SEM investigations revealed typical features induced by the apoptotic pathway. Certainly, this information will be useful increasing scientific the interest in Hys as a natural source of bioactive molecules. Future studies involving assays on pure compounds will be also conducted with the aim to establish a closer correlation between chemical composition and activity. Furthermore, further investigations will be needed to better understand the applications and uses of these EOs and Hys in various fields such as pharmaceutical ones.

## Figures and Tables

**Table 1:** Chemical composition (percentages mean values ± standard deviation) of hop and hemp EO.

N.	COMPONENT ^1^	LRI ^2^	LRI ^3^	Hop EO ^4^	Hemp EO ^5^
1	*α*-thujene	821	823	-	tr
2	*α*-pinene	941	943	tr	8.6 ± 0.03
3	camphene	948	946	-	0.1 ± 0.01
4	*β*-thujene	960	968	-	tr
5	*β*-myrcene	990	987	9.4 ± 0.04	12.4 ± 0.02
6	propanoic acid, 2-methyl-, 2-methylbutyl ester	991	989	1.1 ± 0.02	-
7	propanoic acid, 2-methyl-3-methylbutyl ester	993	996	0.4 ± 0.02	-
8	*α*-phellandrene	1006	1005	-	0.2 ± 0.02
9	3-carene	1010	1008	-	0.3 ± 0.02
10	heptanoic acid, methyl ester	1011	1008	0.2 ± 0.02	-
11	*α*-terpinene	1014	1010	-	0.2 ± 0.03
12	*p*-cymene	1018	1016	-	0.1 ± 0.01
13	limonene	1021	1023	0.4 ± 0.02	27.9 ± 0.02
14	*cis*-*β*-ocimene	1036	1037	-	2.1 ± 0.02
15	*γ*-terpinene	1056	1054	-	0.2 ± 0.02
16	*cis*-sabinene hydrate	1063	1059	-	0.1 ± 0.01
17	terpinolene	1072	1080	-	6.2 ± 0.02
18	linalool	1094	1095	1.3 ± 0.02	-
19	fenchol	1096	1098	-	0.2 ± 0.02
20	octanoic acid, methyl ester	1118	1122	0.2 ± 0.02	-
21	isoborneol	1130	1134	-	tr
22	terpinen-4-ol	1155	1160	-	0.1 ± 0.01
23	octanoic acid	1169	1170	tr	-
24	methyl 6-methyloctanoate	1180	*	0.6 ± 0.02	-
26	*α*-terpineol	1185	1183	0.2 ± 0.02	0.3 ± 0.02
27	nonanoic acid, methyl ester	1226	1224	0.3 ± 0.03	-
28	2-undecanone	1280	1276	0.3 ± 0.02	-
29	4-decenoic acid, methyl ester	1292	*	2.2 ± 0.06	-
30	(*Z*)-methyl geranate	1318	1323	1.2 ± 0.02	-
31	ylangene	1266	1376	0.5 ± 0.03	-
32	*α*-copaene	1385	1392	1.8 ± 0.02	-
33	isocaryophyllene	1398	*	-	0.2 ± 0.02
34	*α*-gurjunene	1418	1420	-	0.2 ± 0.02
35	*β*-caryophyllene	1438	1440	12.2 ± 0.01	22.2 ± 0.06
36	*α*-himachalene	1451	1447	-	0.7 ± 0.03
37	humulene	1466	1473	38.0 ± 0.04	6.5 ± 0.02
38	*β*-eudesmene	1481	1483	4.3 ± 0.03	1.1 ± 0.23
39	*α*-farnesene	1483	1486	-	0.3 ± 0.02
40	*γ*-muurolene	1488	1486	3.6 ± 0.02	-
41	*γ*-gurjunene	1490	1477	-	0.7 ± 0.02
42	*β*-maaliene	1492	*	-	2.3 ± 0.03
43	*α*-selinene	1495	1489	2.1 ± 0.02	-
44	*δ*-cadinene	1510	1511	9.4 ± 0.04	-
45	selina-3,7(11)-diene	1536	1540	1.9 ± 0.02	0.6 ± 0.03
46	caryophyllenyl alcohol	1545	*	0.5 ± 0.03	-
47	caryophyllene oxide	1586	1583	0.7 ± 0.02	2.7 ± 0.03
48	humulene epoxide II	1601	1606	1.0 ± 0.06	0.7 ± 0.02
49	*α*-eudesmol	1611	1615	1.4 ± 0.02	-
50	cedr-8-en-15-ol	1642	*	-	0.6 ± 0.03
51	ledene oxide II	1450	*	-	0.7 ± 0.02
52	*α*-bisabolol	1671	1665	-	0.6 ± 0.02
53	*τ*-cadinol	1638	1630	4.5 ± 0.02	-
54	eudesm-7(11)-en-4-ol	1682	1678	0.1 ± 0.01	0.4 ± 0.04
	SUM			99.8	99.7
	Monoterpenoids			14.8	59.0
	Sesquiterpenoids			79.7	40.5
	Others			5.3	-

^1^ The components are reported according to their elution order on apolar column ^2^ Linear retention indices measured on apolar column; ^3^ Linear retention indices from literature; * LRI not available; ^4^ Percentage mean values of Chinook hop EO components; ^5^ Percentage mean values of kompolti hemp EO components; - Not detected; tr: traces (mean value < 0.1%).

**Table 2:** Chemical composition (percentages mean values ± standard deviation) of hop and hemp Hy.

N.	COMPONENT ^1^	LRI ^2^	LRI ^3^	Hop Hy ^4^	Hemp Hy ^5^
1	butanal, 3-methyl-	648	650	-	2.2 ± 0.02
2	butanal, 2-methyl-	665	662	-	1.2 ± 0.03
3	1-butanol, 2-methyl-	747	744	1.0 ± 0.02	-
4	2-butanal, 3-methyl-	752	748.4	0.8 ± 0.02	-
5	hexanal	802	800	1.1 ± 0.03	0.3 ± 0.02
6	butanoic acid, 3-methyl	842	839	1.6 ± 0.02	-
7	1-hexanol	871	868	-	1.0 ± 0.04
8	2-octen-4-one	875	*	3.3 ± 0.03	-
9	3-heptanone	890	885	0.9 ± 0.02	-
10	1-hepten-6-one	991	987	3.2 ± 0.03	-
11	*p*-cymene	1018	1016	-	0.2 ± 0.02
12	1,8-cineole	1026	1025	-	2.0 ± 0.03
13	*cis*-linalooloxide	1064	1066	4.0 ± 0.01	-
14	*trans*-linalooloxide	1078	1082	1.1 ± 0.03	-
15	*p*-cymenene	1088	1091	-	4.3 ± 0.02
16	linalool	1091	1095	21.2 ± 0.04	6.2 ± 0.02
17	myrcenol	1095	1097	0.5 ± 0.02	-
18	fenchol	1096	1098	-	4.4 ± 0.02
19	1,3,8-*p*-menthatriene	1107	*	-	0.7 ± 0.02
20	isoborneol	1130	1134	2.7 ± 0.02	2.5 ± 0.02
21	*cis*-*p*-mentha-2,8-dien-1-ol	1141	1138	-	25.7 ± 0.02
22	terpinen-4-ol	1155	1160	1.1 ± 0.02	18.1 ± 0.02
23	*α*-terpineol	1185	1183	12.7 ± 0.03	31.2 ± 0.04
24	*cis*-geraniol	1241	1236	5.1 ± 0.02	-
25	caryophyllenyl alcohol	1545	*	2.4 ± 0.03	-
26	caryophyllene oxide	1586	1583	9.0 ± 0.02	-
27	*τ*-cadinol	1638	1460	22.9 ± 0.02	-
28	germacrene D	1451	1477	1.2 ± 0.04	-
29	*α*-eudesmol	1611	1615	4.2 ± 0.02	-
	SUM			100.0	100.0
	Monoterpenoids			48.4	95.3
	Sesquiterpenoids			39.7	-
	Others			11.9	4.7

^1^ The components are reported according to their elution order on apolar column ^2^ Linear retention indices measured on apolar column; ^3^ Linear retention indices from literature; * LRI not available; ^4^ Percentage mean values of Chinook hop EO components; ^5^ Percentage mean values of kompolti hemp EO components; - Not detected; tr: traces (mean value < 0.1%).

**Table 3:** EC_50_ of Kompolti hemp and Chinook hop EOs obtained by dose-dependent MTT assay 48 h of treatments in human cancerous and non-cancerous cell lines. The values are expressed in mg/mL as mean ± standard error mean (sem). SI: Selectivity Index.

EO		HL60	SH-SY5Y	MCF-7	MDA	MCF-10A
Kompolti hemp	EC_50_ mg/mL (×10^−3^)	10.49 ± 1.37	95.87 ± 7.10	241.10 ± 5.23	230.17 ± 9.80	210.97 ± 2.31
	SI	20.11	2.20	0.88	0.92	-
Chinook hop	EC_50_ mg/mL (×10^−3^)	38.92 ± 3.10	315.60 ± 1.92	237.20 ± 2.51	317.51 ± 2.76	294.71 ± 4.96
	SI	7.57	0.93	1.24	0.93	-

HL60 (human acute promyelocytic leukemia cells); SH-SY5Y (human neuroblastoma cells); MCF7 (human metastatic adenocarcinoma breast cells); MDA (human adenocarcinoma breast cells); MCF10A (normal breast epithelial cell).

**Table 4:** EC_50_ of Kompolti hemp and Chinook hop Hys obtained by dose-dependent MTT assay 48 h of treatments in human cancerous and non-cancerous cell lines. The values are expressed in mg/mL as mean ± standard error mean (sem). SI: Selectivity Index.

Hy		HL60	SH-SY5Y	MCF-7	MDA	MCF-10A
Kompolti hemp	EC_50_ mg/mL	6.05 ± 1.54	4.90 ± 0.82	291.96 ± 6.82	127.53 ± 4.77	225.47 ± 6.92
	SI	37.25	45.99	0.77	1.77	-
Chinook hop	EC_50_ mg/mL	10.46 ± 0.52	4.49 ± 0.58	326.76 ± 2.68	140.81 ± 4.72	163.92 ± 3.73
	SI	15.67	36.53	0.50	1.16	-

HL60 (human acute promyelocytic leukemia cells); SH-SY5Y (human neuroblastoma cells); MCF7 (human metastatic adenocarcinoma breast cells); MDA (human adenocarcinoma breast cells); MCF10A (normal breast epithelial cell).

## Data Availability

Data is contained within the article.
